# Genome Sequencing and Carbohydrate-Active Enzyme (CAZyme) Repertoire of the White Rot Fungus *Flammulina elastica*

**DOI:** 10.3390/ijms19082379

**Published:** 2018-08-13

**Authors:** Young-Jin Park, Yong-Un Jeong, Won-Sik Kong

**Affiliations:** 1 Department of Biomedical Chemistry, Research Institute for Biomedical & Health Science, College of Biomedical and Health Science, Konkuk University, 268 Chungwon-daero, Chungju-si 27478, Korea; s11034@kku.ac.kr; 2 Mushroom Research Division, National Institute of Horticultural and Herbal Science, Rural Development Administration, 92, Bisan-ro, Eumseong-gun 27709, Korea; wskong@korea.kr

**Keywords:** carbohydrate active enzyme, *Flammulina elastica*, whole genome sequencing

## Abstract

Next-generation sequencing (NGS) of the *Flammulina elastica* (wood-rotting basidiomycete) genome was performed to identify carbohydrate-active enzymes (CAZymes). The resulting assembly (31 kmer) revealed a total length of 35,045,521 bp (49.7% GC content). Using the AUGUSTUS tool, 12,536 total gene structures were predicted by ab initio gene prediction. An analysis of orthologs revealed that 6806 groups contained at least one *F. elastica* protein. Among the 12,536 predicted genes, *F. elastica* contained 24 species-specific genes, of which 17 genes were paralogous. CAZymes are divided into five classes: glycoside hydrolases (GHs), carbohydrate esterases (CEs), polysaccharide lyases (PLs), glycosyltransferases (GTs), and auxiliary activities (AA). In the present study, annotation of the predicted amino acid sequences from *F. elastica* genes using the dbCAN CAZyme database revealed 508 CAZymes, including 82 AAs, 218 GHs, 89 GTs, 18 PLs, 59 CEs, and 42 carbohydrate binding modules in the *F. elastica* genome. Although the CAZyme repertoire of *F. elastica* was similar to those of other fungal species, the total number of GTs in *F. elastica* was larger than those of other basidiomycetes. This genome information elucidates newly identified wood-degrading machinery in *F. elastica*, offers opportunities to better understand this fungus, and presents possibilities for more detailed studies on lignocellulosic biomass degradation that may lead to future biotechnological and industrial applications.

## 1. Introduction

*Flammulina elastica* (*Physalgacriaceae*; white-rotting basidiomycete) was first recognized in 1999 by Redhead and Petersen [1]; however, little is known about its biology, including its molecular characteristics. Recently, *F. elastica* spores were reported to differ from those of *Flammulina velutipes* with SQ = 2.5–3 (the ratio of length and width of the spores) [2]. In addition, Ripková et al. [3] reported that *F. elastica* had similar morphological characteristics to *F. velutipes*, but some specimens identified morphologically as *F. velutipes* had *F. elastica* internal transcribed spacer (ITS) sequences. Furthermore, *F. elastica* was found to be basal to *F. velutipes* based on a phylogenetic analysis of ITS DNA sequences. These discoveries indicated that further investigations were needed to resolve these discrepancies for morphological and molecular delimitation. Macromorphological characters and habitat are also important for identifying *Flammulina* species. Based on its habitat, *F. elastica* is generally considered lignicolous, with direct growth from wood [4]. 

Basidiomycetes can efficiently degrade lignocellulosic biomass, especially that derived from plants, because of their diverse CAZymes [5,6]. This ability allows the fungi to inhabit and colonize a variety of natural environments, such as softwoods, hardwoods, grasses, crops, and forest waste. Therefore, they are frequently found in nature. Wood-rotting fungi are generally divided into two groups: white rot fungi and brown rot fungi. White rot fungi, representing more than 90% of wood-rotting basidiomycetes, can degrade both lignin and polysaccharides, resulting in a white or yellowish color in residual wood [6,7]. An understanding of lignocellulosic biomass degradation mechanisms of basidiomycetes will allow us to use this process for appropriate applications. 

Enzymes involved in the biosynthesis, modification, binding and catabolism of carbohydrates are known as carbohydrate-active enzymes (CAZymes). These CAZymes are divided into several classes, including glycoside hydrolases (GHs), carbohydrate esterases (CEs), polysaccharide lyases (PLs), glycosyltransferases (GTs), and auxiliary activities (AA), based on their catalytic activities. They are further classified into several families, based on their functional amino acid sequences and structural similarities (8, CAZy database; http://www.cazy.org/). These CAZymes have received attention because of their biotechnological and industrial applications. Precursors produced by these enzymes can be used to generate bio-based products, such as food, paper, textile, animal feed, and other chemicals, including biofuels [6,7].

We previously reported the genome sequence of *F. velutipes*; we also found a well-developed wood-degrading machinery, with the identification of various CAZymes in the genome [8]. To date, several genome-sequencing studies have been performed to reveal the genes encoding biomass-degrading enzymes [6]. For instance, genome sequencing of the white rot basidiomycete *Phanerochaete chrysosporium* revealed a larger repertoire of plant biomass-degrading enzymes than that of *Ustilago maydis* (a biotrophic phytopathogen) [6,9,10]. Furthermore, in the post-genomic era, studies of biomass-degrading enzymes represent a major area of research to understand wood-degrading machinery and describe the CAZyme repertoires of fungal species. Here, for the first time, we report the genome sequence of *F. elastica*. The aim of this study was to identify CAZyme genes in the genome of the white rot fungus *F. elastica* to increase the applicability of these biotechnologically and industrially useful enzymes. This information on the genome of *F. elastica*, as well as genes predicted to encode CAZymes in this organism, will facilitate our understanding of this white rot fungus that possesses enormous potential for biotechnological and industrial applications.

## 2. Results and Discussion

### 2.1. Genome Sequence Assembly, Gene Modeling, and Genome Comparisons

The genomic DNA library of *F. elastica* strain KACC46182 was sequenced with 100 bp paired end reads on a single flow cell lane on a HiSeq 2000 platform (Illumina Korea, Seoul, Korea). The short reads (total of 28,829,056; 100 bp paired-end reads) were processed using the Trimmomatic tool for quality control and adapter trimming. The resulting short reads (26,777,626 reads, > Q 30) were analyzed for de novo assembly using the Velvet assembly tool with a kmer-size search range of 17–31. The optimized assembly (31 kmer) that resulted consisted of 13,877 sequence contigs with a total length of 35,045,521 bp (49.7% GC contents) and N50 length of 42,684 bp. The sequence contigs were processed using the AUGUSTUS tool for ab initio gene prediction. A total of 12,536 gene structures were predicted, with an average gene length of 1973 bp (Table 1). The average exon and intron lengths were 233.91 and 62.29 nucleotides, respectively. The general features of the *F. elastica* strain KACC46182 genome, based on the assembly and gene model statistics, are presented in Table 1. Of the 12,536 predicted genes, 84% (10,523) had significant sequence similarity (0.001 > e-value) with documented proteins in NCBI-NR (Table S1). In addition, BLASTP searches against the NCBI fungal genome database revealed that 10,088 (80.5%) of the predicted proteins shared sequence similarity with predicted proteins in documented fungal sequences (Table S2). 

The total number of genes in *F. elastica* was comparable to that of its nearest sequenced species, *F. velutipes* [11], as well as to those of other basidiomycetes with a similar genome size (Table 2). In addition, 272 transfer RNA (tRNA) genes in the *F. elastica* genome were identified by tRNAscan-SE [12] (Table S3). In a protein family search against the Pfam 31.0 database, 6829 genes and 1911 genes were annotated as functional proteins and multi-domain protein families, respectively (Table S4).

**Figure 1:**
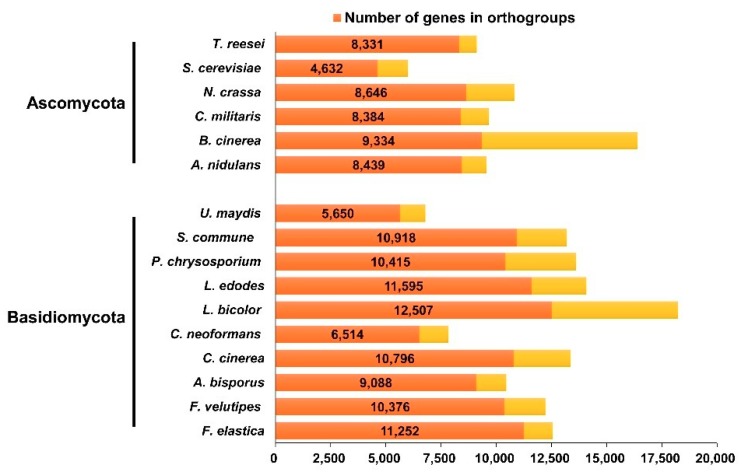
Number of genes and orthologs in *F. elastica* and other fungal species.

**Figure 2:**
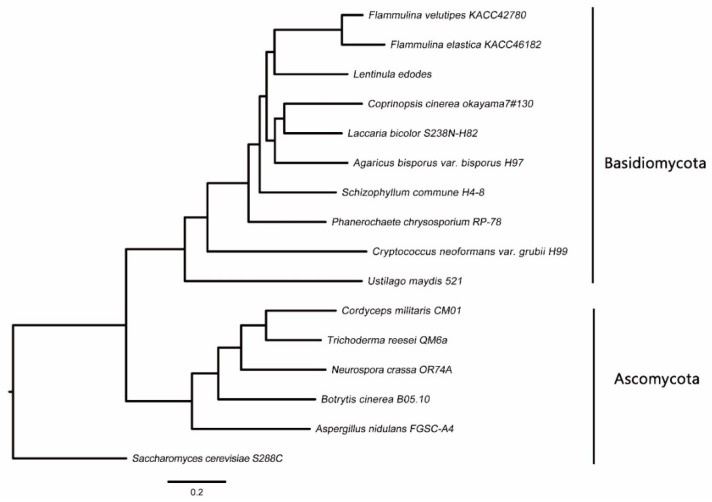
Phylogenetic tree of fungal species based on ortholog clustering.

Through a cluster analysis with other sequenced fungal species, 6806 groups containing at least one *F. elastica* protein were identified (Table S5). Analysis of these clusters suggested that 57.8% of *F. elastica* proteins had orthologs in the Dikarya, and thus were conserved in basidiomycetes and ascomycetes (Figure 1 and Table S5). Among the set of homologous genes, there were 582 single copy orthologs. *F. elastica* contained 24 species-specific genes, of which 17 genes were paralogous. As shown in Figure 2, *F. elastica* was classified into one group with *F. velutipes* by ortholog-based clustering analysis.

### 2.2. F. elastica CAZymes and Genome-Wide Comparisons with other Fungal Species

**Figure 3:**
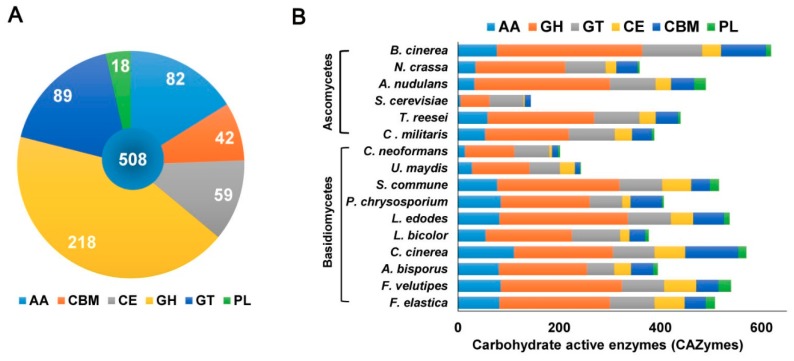
Carbohydrate-active enzymes in (**A**) the *F. elastica* genome and (**B**) other fungal species. AA, auxiliary activities; GH, glycoside hydrolase; GT, glycosyltransferase; CBM, carbohydrates- binding module; PL, polysaccharide lyase.

In the present study, the genome sequence of *F. elastica* revealed several genes associated with assembly (GT) and breakdown (GHs, PLs, CEs) of carbohydrate complexes. In addition, the *F. elastica* genome was found to contain a vast array of genes coding for initial lignin degradation (auxiliary activities; AA), as well as a carbohydrate-binding module (CBM). Annotation of the predicted amino acid sequences of *F. elastica* genes using the dbCAN CAZyme database revealed 508 CAZymes, including 82 AAs, 218 GHs, 89 GTs, 18 PLs, 59 CEs, and 42 CBMs in the *F. elastica* genome (Figure 3A and Table S6). For genome-wide comparisons, amino acid sequences of 7 fungal species were also annotated using the HMMER 3.0 package (http://hmmer.org/) with the dbCAN CAZyme database (http://csbl.bmb.uga.edu/dbCAN/) [13]. In addition, annotated CAZymes of eight other fungal species were obtained from the CAZy database (8, CAZy database; http://www.cazy.org/) and JGI Fungi Portal database (https://genome.jgi.doe.gov/programs/fungi/index.jsf). Figure 3B shows the distribution of CAZymes in *F. elastica* and 15 other fungal species genomes (see also Table S7).

#### 2.2.1. Glycosyltransferases (GTs)

GTs (EC 2.4.x.y) are enzymes that catalyze the formation of glycosidic linkages to form glycosides, which are involved in the biosynthesis of oligosaccharides, polysaccharides, and glycoconjugates [14,15]. These enzymes utilize activated donor sugar phosphates and catalyze glycosyl group transfer to specific acceptor molecules to form glycosidic bonds [15,16,17]. 

**Figure 4:**
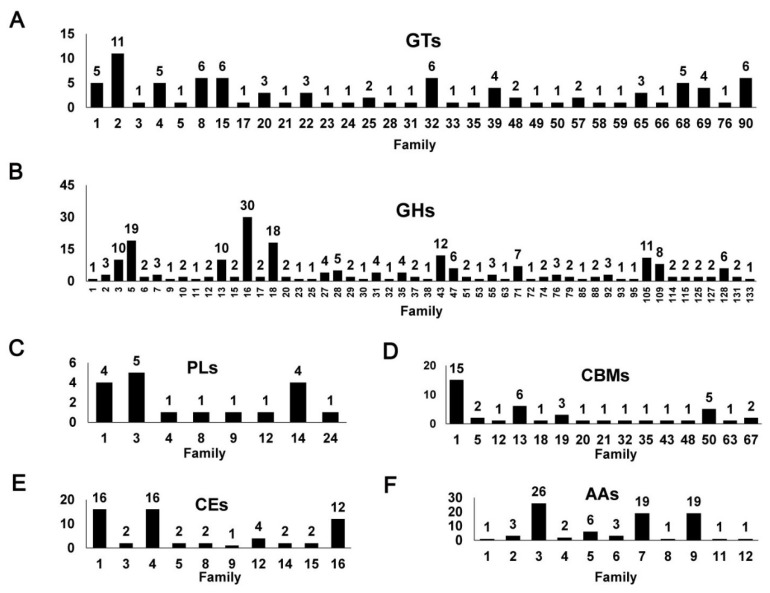
Number of CAZymes in *F. elastica*. Number of (**A**) GT families; (**B**) GH families; (**C**) PL families; (**D**) CBM families; (**E**) CE families; (**F**) AA families. AA, Auxiliary Activities; GH, glycoside hydrolase; GT, glycosyltransferase; CBM, carbohydrates- binding module; PL, polysaccharide lyase.

**Figure 5:**
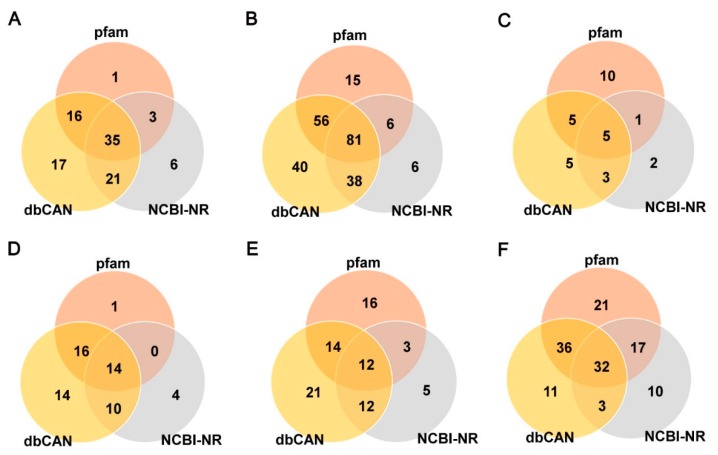
Venn diagrams of CAZymes predicted in *F. elastica* by three different database searches. (**A**) GT families; (**B**) GH families; (**C**) PL families; (d) CBM families; (**E**) CE families; (**F**) AA families. Pfam, protein family database (Pfam 31.0, http://pfam.xfam.org); dbCAN, CAZyme database (http://csbl.bmb.uga.edu/dbCAN/); NCBI-NR, National Center for Biotechnology Information (NCBI) non-redundant database.

CAZyme annotation revealed that *F. elastica* contains a total of 32 GT families in its genome sequence, of which 50% (16 families) with only one gene were identified, based on a dbCAN database search (Figure 4A and Table S8). In addition, 65 and 55 genes predicted to encode GTs were identified by BLASTP (NCBI-NR) and a protein family database (Pfam 31.0) search, respectively (Tables S1 and S4), and 35 genes predicted to encode GTs based on all three different databases were identified (Figure 5A and Table S9). Among the 99 genes predicted to encode GTs based on one of the three different databases, 17, 6, and 1 genes predicted to encode GTs were uniquely identified based on dbCAN, NCBI-NR, and Pfam database searches, respectively (Figure 5A and Table S9). 

GTs were classified into families based on amino acid sequence similarities [16,17]. However, functional prediction of putative GTs based on sequence homology is uncertain, because many GTs have different activities, even though GT sequences are highly similar. In large polyspecific families, such as GT2 or GT4, sequence similarities are restricted to only a portion of the catalytic domain, whereas in monospecific families, they are generally observed for the entire catalytic domain [14]. Therefore, even if amino acid sequences are highly similar within a polyspecific family, their functions cannot be precisely determined based on sequence similarity alone [14]. 

**Figure 6:**
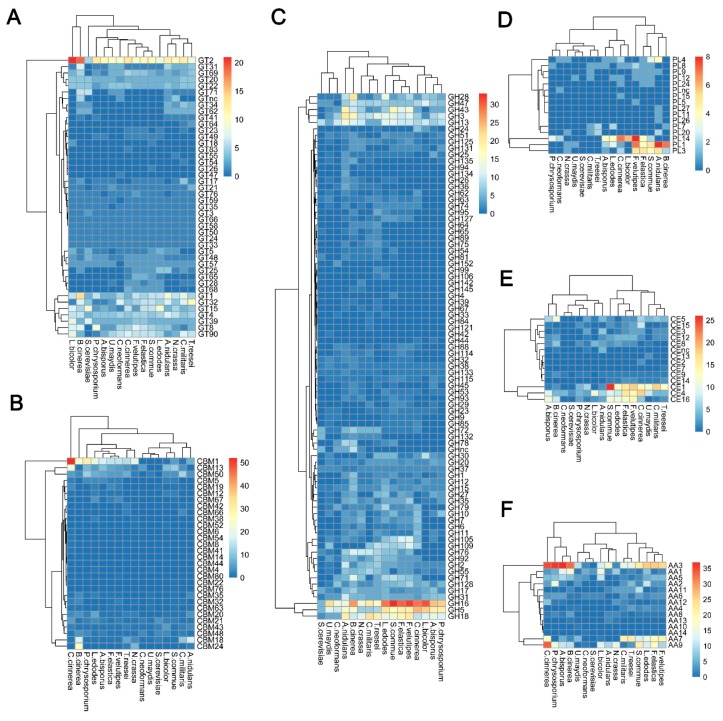
Distribution of CAZymes in *F. elastica* and other fungal species. (**A**) GT families; (**B**) CBM families; (**C**) GH families; (**C**) PL families; (**E**) CE families; (**F**) AA families.

Completely sequenced organisms, including archaeal, bacterial, or eukaryotic organisms, show that a large number of GTs (about 1–2% of gene products) are encoded by their genomes (8, CAZy database; http://www.cazy.org/). Among the GT families listed in the CAZy database, two families, GT2 and GT4, account for about half of the total number of GTs. In this study, among the predicted GTs in the *F. elastica* genome, the GT2 family with 11 genes was prominent (Figure 4A and Table S8). GT2 has been reported to act as a cellulose synthase, chitin synthase, galactosyltransferase, glucosyltransferase, mannosyltransferase, and rhamnosyltransferase, among other enzymes [14]. Indeed, based on NCBI fungal genome database searches, some of these GT2 family genes were annotated as chitin synthase (EC 2.4.1.16) and dolichyl-phosphate β-d-mannosyltransferase (EC 2.4.1.83) involved in fungal cell wall biosynthesis and *n*-glycan biosynthesis, respectively (Tables S1 and S2). Genome-wide comparisons confirmed that the GT2 family was prominent, suggesting that the GT2 family is a major component of GT families in most fungal species. A number of GT2 families have been identified in 16 fungal genomes, including nine species of basidiomycetes and four species of ascomycetes, respectively (Figure 6A and Table S8). Differences, specifically number and function, in GTs were observed among families in a public database [14]. In addition, Breton et al. [14] indicated that not all sequences encoding GT were present in the database, and the number of families is going to increase with the incorporation of newly discovered GT genes. At the time of writing (May 2018), the database comprised more than 410,000 classified and 8800 non-classified GT sequences divided into 105 families (8, CAZy database; http://www.cazy.org/). Of GT family members, more than 126,000 sequences from archaea, bacteria, eukaryote, and viruses were classified into the GT2 family in databases until recently (8, CAZy database; http://www.cazy.org/).

GTs are resident membrane proteins of the endoplasmic reticulum and Golgi apparatus. All GT proteins have large C-terminal catalytic domains, a short N-terminal cytoplasmic tail, and a signal-anchor domain (16–20 amino acids) [18]. Signal-anchor domains act as both transmembrane regions and uncleavable signal peptides [19]. The difference between signal peptides and signal anchors seems to be the length of the hydrophobic domain [20,21]. Signal peptide prediction revealed six genes comprising the signal peptides in 99 GT genes in *F. elastica* (Table S10). These six genes showed positive peaks (hydrophobic) in hydropathy profiles in 16–20 amino acid regions. These results suggest that the predicted signal peptide sites in six genes are uncleavable and that these regions likely act as the signal-anchor domains.

Previous studies have described the difficulty of identification and classification of GTs based on sequence similarity; therefore, a GT identification method that does not rely solely on sequence similarity is required, for example, the development of a computational method to identify the transmembrane region of Golgi-localized signal-anchor-type GTs and discover novel GTs [22]. Furthermore, additional studies based on structural, modeling, and mutational analyses are needed to elucidate enzyme characteristics and function.

#### 2.2.2. Glycoside Hydrolases (GHs)

GHs (glycosidases or glycosyl hydrolases, EC 3.2.1.-) are enzymes that catalyze the hydrolysis of glycosidic bonds of complex carbohydrates and key enzymes involved in carbohydrate metabolism. In addition, GHs are common enzymes in nature that degrade the most abundant biomasses, such as cellulose, hemicellulose, and starch [23,24]. 

GHs can be assigned to various families using algorithmic methods based on sequence similarity. Henrissat [24] conducted comparisons of 301 amino acid sequences of GHs and classified 291 sequences into 35 families. At the time of writing (May 2018), the CAZy database comprised more than 487,000 classified and 8700 non-classified GH sequences that were divided into 153 families (8, CAZy database; http://www.cazy.org/). In the present study, a total of 218 GHs classified into 52 families were predicted in the *F. elastica* genome based on a dbCAN database search (Figure 4B and Table S8). GH family classification also revealed that 15 families consisted of only one gene and that GH16 was prominent among 30 genes (Figure 4B). In addition, 131 and 158 genes predicted to encode GHs were identified by BLASTP (NCBI-NR) and protein family database Pfam 31.0 searches, respectively (Tables S1 and S4), and 81 genes predicted to encode GHs were identified using three different databases (Figure 5B and Table S11). Among them, 40, 6, and 15 genes predicted to encode GHs were uniquely identified by a dbCAN database, BLASTP (NCBI-NR) and protein family (Pfam 31.0 database) searches, respectively. In genome comparisons, the GH16 family was also prominent in 14 to 33 other fungal species, except for some ascomycetes, including *Aspergillus nudulans* [25], *Cordyceps militaris* [26], *Saccharomyces cerevisiae* [27], and *Trichoderma reesei* [28] (Figure 6C and Table S8). In addition, multiple copies of GH5 and GH18 in *F. elastica* were similar to those in other basidiomycetes. 

GH family 16 comprises a number of enzymes with known activities. These enzymes include lichenase (EC 3.2.1.73), xyloglucan xyloglucosyltransferase (EC 2.4.1.207), agarase (EC 3.2.1.81), κ-carrageenase (EC 3.2.1.83), endo-β-1,3-glucanase (EC 3.2.1.39), endo-β-1,3-1,4-glucanase (EC 3.2.1.6), and endo-β-galactosidase (EC 3.2.1.103). Most of the enzymes in the GH16 family contain the conserved motif Glu-Xaa-Asp-Xaa-(Xaa)-Glu (EXDX[X]E) in their amino acid sequences. The first and the last glutamic acid (E) residue functions as a nucleophile and Brønsted acid/base, respectively (Figure S1A) [29,30]. All of the predicted GH16 family members in *F. elastica* also showed this conserved motif, except for one GH16 family member, which showed the motif Glu-Xaa-Val-Xaa-(Xaa)-Glu (EXVXXE) (Figure S1A). Among the GH16 family members, nine genes showed the catalytic motif Glu-Ile-Asp-Ile-Ile-Glu (EIDIIE). Kotake et al. [31] showed that glutamic acid (Glu, E) residues at both the first and last positions of the motif are important for the catalytic activity of GH16 family enzymes.

Signal peptide prediction revealed about half of the total number of GH genes (94 out of 218 GHs) comprising signal peptides in *F. elastica* (Table S10). Many GHs have a signal sequence, since they are secreted or targeted at other cellular locations, such as the periplasmic space or Golgi body. However, not all glycosyl hydrolases have signal sequences in their genes. Approximately two-thirds of all GH genes have a signal sequence, whereas one-third of genes have no signal sequence, suggesting their cellular location [32]. 

Substrate specificity is one of the distinctive features of enzymes: GH5, -6, -7, -8, -9, -12, -44, -45, and -48 (cellulases) family members, biochemically characterized proteins, are active against cellulose; and GH10, -11, and -30 family members (xylanases) are active against xylose; GH18, -19, and -85 family members (chitinases) are active against chitin [8,33]. In this study, CAZyme annotation revealed that *F. elastica* contains a series of genes associated with cellulase (GH5, -6, -7, -9, and -12), xylanase (GH10, -11, and -30), and chitinase (GH18 and -85) in its genome sequence (Figure 4B and Table S8). GHs are essential for the processing of polysaccharides such as plant cellulose and xylan, which represent a major source of carbon in nature. Chitin is also an important carbon and nitrogen source in ecosystems (8, CAZy database; http://www.cazy.org/). Synergistic action of many enzymes is required to degrade such polysaccharides. Polysaccharides can be degraded to short oligosaccharides by the synergistic activities of GHs by the endo-mode of action and exo-mode of action of Ghs such as endo-cellulase and endo-cellulase, respectively. β-glucosidases (EC 3.2.1.21) are also members of GH families (GH1 and GH3) that convert cellobiose into glucose. Most enzymes involved in polysaccharide degradation are classified into several GH families [8,34]. CAZyme annotation revealed that *F. elastica* also possesses other GH family members, including 1 GH1 and 10 GH3 in its genome (Figure 4B and Table S8). Polysaccharides such as cellulose and xylan in plant cell walls often form complex structures. Thus, synergistic activities of other GHs are required to degrade these complexes. Fungi play an important role in the hydrolysis of cellulose, xylan, and chitin in the environment and thus have potential uses in biotechnology. 

Recently, sequenced bacterial genomes have revealed the variability of GHs involved in cellulose, chitin, and xylan degradation and their potential for industrial degradation of biopolymers [35,36,37]. In addition, fungi also show high levels of hydrolytic activity involved in polysaccharide degradation in nature, and the degrading machineries of many species have been characterized for their potential in biotechnological applications [33,38,39]. In the present study, CAZyme annotation revealed that *F. elestica* showed strong potential for biotechnological applications, encoding more than 200 genes for various GHs that target a broad range of possible substrates, such as polysaccharides.

#### 2.2.3. Polysaccharide Lyases (PLs)

Polysaccharides are frequently found in nature. These are essential cellular components of all living organisms, ranging from bacteriophages to higher eukaryotes [40]. PLs, also known as eliminases, are enzymes (EC 4.2.2.-) that cleave uronic acid-containing polysaccharides through a β-elimination mechanism, rather than via hydrolysis, to produce unsaturated polysaccharides [41]. PLs are classified into families based on recognizable sequence homologies (8, CAZy database; http://www.cazy.org/). Until recently, PLs have been classified into 28 families, with more than 13,500 classified and 1200 non-classified PL sequences in the CAZy database (8, CAZy database; http://www.cazy.org/). Our results showed that a total of 18 PLs classified into eight families were predicted in the *F. elastica* genome based on a dbCAN database search (Figure 4C and Table S8). Among them, the PL3 family was prominent, and five families, including PL5, -8, -9, -12, and -24, consisted of only one PL (Figure 4C and Table S8). Additionally, 11 and 21 genes predicted to encode PLs were identified by BLASTP (NCBI-NR) and protein family database (Pfam 31.0) searches, respectively (Tables S1 and S4), and five genes predicted to encode PLs were identified using all three different databases (Figure 5C and Table S12). Five, two, and 10 genes predicted to encode PLs were uniquely identified by dbCAN, NCBI-NR, and Pfam database searches, respectively (Figure 5C and Table S12). Our results showed that other basidiomycetes, except for *F. elastica* and *U. maydis* [10], had high numbers of genes encoding PL14 family members in their genomes and that there were no PL14 family members in ascomycetes (Figure 6D and Table S8). The distribution of some PL family members appeared to be phylum specific. For instance, PL10 and PL11 were only found in ascomycetes, whereas PL15 appeared to be specific to the Basidiomycota [42]. Likewise, in the present study, PL family members 11 and 15 were found only in *A. nidulans* (ascomycete) [25] and *Coprinopsis cinerea* (basidiomycete) [43], respectively (Table S8). In addition, PL5, -14, -15, and -24 family members are Basidiomycota specific, although, except for PL14 family members, they are present only in a few basidiomycetes.

Pectate/pectin, an acidic polysaccharide in plant cell walls, is less prominent in plant biomass than cellulose and hemicellulose [44,45]. Pectate and pectin are partially branched polymers containing homocopolymeric blocks (1→4 linked α-d-galacturonate) and homopolymeric blocks (1→4 linked α-d-methylgalacturonate), respectively [46]. The enzymes that degrade polygalacturonan (PGA), smooth regions of polysaccharides, are designated pectate or pectin lyases [45]. Pectate lyases and pectin lyases are mainly produced by bacterial species and fungal species, respectively. However, fungal species also produce pectate lyases, which are often accompanied by other lyases and hydrolases to act on pectin and/or pectate [46]. Pectin and pectate lyases have been classified into six PL families, namely PL1, -2, -3, -9, -10, and -22, found in the CAZy database (8, CAZy database; http://www.cazy.org/). To date, all characterized pectin lyases (EC 4.2.2.10) belong to the PL1 family, and the fungal pectate lyases (EC 4.2.2.2 and EC 4.2.2.9) belong to families PL1, PL3, and PL9 (8, CAZy database; http://www.cazy.org/). Our results also showed that genes encoding PL family members, including families 2, 10, and 22, were not found in the *F. elastica* genome or in other fugal species analyzed in this study (Table S8). Moreover, the majority of PLs were pectate lyases, such as members of PL families 1 and 3 in *F. elastica* and other fungal species, including *F. velutipes* [11], *S. commune* [47], *A. nidulans* [25], and *B. cinerea* [48]. In contrast, most fungal species lack PL family member 9, which has been found in three basidiomycetes (*F. elastica*, *F. velutipes*, and *Schizophyllum commune*) and in two ascomycetes (*A. nidulans* and *C. militaris*) [25,26] (Figure 6D and Table S8). Signal peptide prediction revealed 13 out of 18 PLs harbored a signal sequence in their genes, and PL1 and -3 were the most abundant families with members containing signal peptides. Additionally, 13 PLs contained a signal peptide but have no transmembrane domains, suggesting that these PLs are secreted (Table S10). 

As yet, biochemical information on the enzymes that degrade pectin or pectate in basidiomycetes is relatively scarce compared with that of other bacterial and fungal species. However, to date, the genomes of many basidiomycetes have been sequenced, revealing many genes that encode CAZymes, including PLs, and that have the potential to be used in biotechnological applications. Furthermore, there is great potential to find a novel PL with unique properties in basidiomycetes, because of their diverse ecological roles and variety of genes encoding putative pectinases in their genomes. For instance, *S. commune* [47], one of the most efficient (hemi) cellulose degrading-basidiomycetes, has a wealth of putative pectin-degrading lyases and therefore produces high levels of pectinases [47,49]. In addition, this basidiomycete has been shown to produce higher levels of polygalacturonase than *Aspergillus niger* in wheat bran cultures [49]. Although enzymatic characterization was not explicitly performed in this study, there were similar numbers of genes encoding PL family members 1, 3, and 9 in the *F. elastica* genome as those in *S. commune* [47], suggesting that *F. elastica* might be a candidate for future studies focused on polysaccharide lyases and their biotechnological applications.

#### 2.2.4. Carbohydrate-Binding Modules (CBMs)

Amino acid sequences having carbohydrate-binding activity within a carbohydrate-active enzyme are designated CBMs, which fold into structurally discrete modules [50,51]. Generally, CBMs bind to carbohydrate ligands and enhance the catalytic efficiency of carbohydrate-active enzymes [50]. 

CBMs are most commonly associated with GHs. They have also been found in several PLs and GTs [52]. In addition, CBMs present in proteins without hydrolytic activity are parts of a scaffolding (scaffoldin) subunit that organizes the catalytic subunits into a non-covalent multi-protein complex called a cellulosome [51]. Enzymatic complexes bearing CBMs show more efficient degradation of substrates, and catalytic efficiency is reduced when CBMs are removed from the scaffolding of cellulosomes [51].

Similar to glycoside hydrolases, CBMs can be classified into families based on amino acid sequence similarity. Until recently, CBMs have been classified into 80 families with more than 127,000 classified and 500 non-classified CBM sequences in the CAZy database (8, CAZy database; http://www.cazy.org/). In the present study, we found that a total of 42 CBMs classified into 15 families were predicted in the *F. elastica* genome based on a dbCAN database search (Figure 4D and Table S8). CBM family 1 was prominent, and five families, including CBM12, -18, -20, -21, -32, -35, -43, -48, and -63, were represented by only one CBM in the *F. elastica* genome based on a dbCAN database search (Figure 4D). Moreover, 16 and 19 genes predicted to encode CBMs were identified by BLASTP (NCBI-NR) and protein family database (Pfam 31.0) searches, respectively (Tables S1 and S4), and two genes predicted to encode CBMs were identified using the three different databases (Figure 5D and Table S13). Among the predicted CBMs, 14, 4, and 1 genes were uniquely identified by dbCAN, NCBI-NR, and Pfam database searches, respectively (Figure 5D and Table S13). Although *F. elastica* does not have unique CBMs in its genome, the distribution of CBMs, with multiple copies of CBM1, -13, and-50 family members, was similar to those found in other fungal species. However, the abundance of some family members differed between basidiomycetes and ascomycetes. Ascomycetes have more CBM family 18 members than other basidiomycetes, and members of CBM families 5 and 12 are not observed in all ascomycetes (Figure 6B and Table S8). These results are consistent with those of a previous study by Zhao et al. [42], which showed that ascomycetes have more members of CBM family 18 but fewer of CBM5 and -12 than basidiomycetes. Interestingly, the distribution of CBMs in fungal species revealed that the highest number of CBMs, 105, including 52 CBM family 1, are found in the genome of the coprophilic fungus *C. cinerea* [43] (Figure 6B). A previous study by Fernandez-Fueyo et al. [53] showed similar results: the *C. cinerea* genome contains a vast array of genes encoding CBMs, with the majority of these belonging to CBM family 1.

CBMs have traditionally been considered essential modules of cellulases, especially cellobiohydrolases, classified into the families GH6 and -7 [54]. Our results identified two genes that encode GH6 and GH7 members and that contain a CBM 1 family member (Table S6). Furthermore, our results revealed several CBM families in genes encoding several CAZymes, including 16 GHs, four CEs, and one AA, implying that these CAZymes may require CBM to efficiently degrade substrates (Table S6).

#### 2.2.5. Carbohydrate Esterases (CEs)

Esterases, which act on ester bonds, are widely used as biocatalysts in industrial processes and biotechnology [55,56]. CEs represent a class of esterases that generally catalyze *O*-de- or *N*-deacylation to remove esters of substituted saccharides [57]. These CEs are classified into 15 families, with more than 54,900 classified and 1200 non-classified CE sequences in the current CAZy database (8, CAZy database; http://www.cazy.org/). CEs show great diversity in substrate specificity, such as specificity for xylan (acetylxylan esterases, EC 3.1.1.72), acetic ester (acetyl esterases, EC 3.1.1.6), chitin (chitin deacetylases, EC 3.5.1.41), peptidoglycan (poly-*N*-acetylglucosamine deacetylases, EC 3.5.1.104), feruloyl-polysaccharide (feruloyl esterases, EC 3.1.1.73), and pectin (pectinesterase, EC 3.1.1.11) [58].

Our results revealed a total of 59 predicted CEs classified into 11 families in the *F. elastica* genome based on a dbCAN database search (Figure 4E and Table S8). CE1 and -4 families were prominent, with 16 CEs, and the CE16 family was the second largest family with 12 CEs in *F. elastica* genome (Figure 4E). However, relatively low numbers of CEs were identified by BLASTP (NCBI-NR) searches, with 32 and 12 genes predicted to encode CEs in the three different databases. In addition, 21, 5, and 16 genes predicted to encode CEs were uniquely identified by dbCAN, NCBI-NR, and Pfam database searches, respectively (Figure 5E and Table S14). Genome-wide comparisons revealed that the total number of CEs in *F. elastica* was similar to those found in other basidiomycetes, including *F. velutipes* [11], *C. cinerea* [43], and *S. commune* [47], with 57 to 63 CEs each (Figure 6E). In addition, CE1, -4, and -16 families are prominent in several basidiomycetes (Figure 6E). Our results showed that CE families vary in abundance among basidiomycetes and ascomycetes. For instance, only five CEs (four CE4 families and one CE9 family) and two CEs (both in family CE4) were found in *Cryptococcus neoformans* [59] and *S. cerevisiae* [27], respectively (Table S8). CAZyme prediction based on the dbCAN database indicated a vast array of genes encoding CE10 family members in the *F. elastica* genome. However, most members of the CE10 family have been found to act on non-carbohydrate substrates [8,60]; therefore, they were not included in this study. Signal peptide prediction revealed that 27 out of the 59 CEs were predicted to harbor signal sequences, and CE4 family members were the most common CEs containing signal peptides. Additionally, these 27 CEs that contained signal peptides had no transmembrane domains, suggesting that these CEs are secreted (Table S10).

Despite a large number of enzymes recently identified and classified as CEs, only a few members of CE families have been biochemically and structurally analyzed. Among these members, several characteristic features in their amino acid sequences have been identified. For instance, members of the CE1, CE4, CE5, and CE7 families of archaeal, bacterial, and eukaryotic origin have been characterized as possessing the Ser-His-Asp catalytic triad, as well as the GXSXG (Gly-Xaa-Ser-Xaa-Gly) conserved motif. CE2 and CE3 family members possess the Gly-Asp-Ser-(Leu) (GDS(L)) motif, rather than the GXSXG conserved motif with the Ser-His catalytic diad and Ser-His-Asp catalytic triad, respectively [61]. CE16 family members also possess the GDS(L) catalytic motif and Ser-Gly-Asn-His (SGNH) catalytic residues. In the present study, several CE family members were found to have conserved motifs, such as GXSXG, in their amino acid sequences (Table S6). Esterases showing high homology to class C β-lactamases and containing a Gly-Xaa-Xaa-Leu (GXXL) motif [62,63] were also identified. Likewise, some CE family members, especially members of the CE16 family, were found to have the (GXXL) motif (Table S6).

CE families generally catalyze *O-*de- or *N*-deacylation to remove the acylated moieties of polysaccharides, facilitating access of GHs to accelerate the degradation of these polymers and assisting in biomass saccharification [64]. Thus, our results demonstrate the extensive range of genes that code for CE family members in the *F. elastica* genome, suggesting the potential for this fungus to be used in biotechnological applications, such as biofuel production.

#### 2.2.6. Auxiliary Activities (AAs)

Members of families GH61 and CBM33 were found to be lytic polysaccharide monooxygenases (LMPOs), resulting in reclassification of these families into a new category in the CAZy database [8,65]. Currently, lignin degradation enzymes such as LMPOs are classified into AA families in the CAZy database, and members of these families are mainly involved in depolymerization of non-carbohydrate structural components (lignin) or found as primary cell wall contents of plants [7]. These AAs are classified into 15 families, with more than 10,300 classified and 100 non-classified AA sequences in the current CAZy database (8, CAZy database; http://www.cazy.org/). In addition, the AA members are presently grouped into eight families of ligninolytic enzymes and three families of lytic polysaccharide monooxygenases. These AA enzymes are classified into families based mainly on amino acid sequence similarities. In the present study, CAZyme annotation revealed that *F. elastica* contains a total of 11 AA families with 82 AAs in its genome sequence (Figure 4F and Table S8). AA family classification also revealed that the majority of AAs are AA3 family members, with 26 AA3 family members (glucose-methanol-choline (GMC) oxidoreductase; alcohol oxidase, aryl-alcohol oxidase/glucose oxidase, cellobiose dehydrogenase, pyranose oxidase), and AA7 (glucooligosaccharide oxidase) and -9 (lytic polysaccharide monooxygenase; GH61) comprising the second largest families, each with 19 AAs encoded in the *F. elastica* genome (Figure 4F). For each family, 62 and 106 genes were predicted as AAs according to BLASTP (NCBI-NR) and protein family database (Pfam 31.0) searches, respectively (Tables S1 and S4), and 32 genes were predicted to encode AAs using all three different databases. Eleven, 10, and 21 genes were predicted to encode AAs by dbCAN, NCBI-NR, and Pfam database searches, respectively (Figure 5F and Table S15). The total number of AAs in the *F. elastica* genome were similar to those in other white rot or white rot-like fungus, such as *F. velutipes* (white rot) [11], *Lentinula edodes* (white rot) [66], and *S. commune* (white rot-like) [47], but not *P. chrysosporium* (white rot) [9] (Table S8). However, the total number of AAs in three fungal species, including *Laccaria bicolor* (ectomycorrhizal fungus) [67], *U. maydis* (plant pathogen) [10], and *C. neoformans* (yeast) [59] was less than that in other sequenced basidiomycetes (Figure 6F and Table S8). To date, unicellular and xerophilic mold-like basidiomycetes such as *C. neoformans* [59], *Rhodotorula glutinis*, and *Wallemia sebi* have also been shown to possess a very limited number of genes coding for polysaccharide degradation enzymes [7]. Additionally, *U. maydis* (a biotrophic plant pathogenic fungus) [10] has been found to possess a minimal set of genes encoding polysaccharide degradation enzymes for defenses against plants [9,10]. In addition, *L. bicolor* [67] has been reported to possess more genes encoding enzymes that modify polysaccharide backbones than genes encoding accessory enzymes. Thus, the most abundant genes code for CAZymes involved in plant cell wall degradation [7].

In early works, several AA families were found to possess conserved motifs necessary for interaction with target substrates. For instance, a laccase (EC 1.10.3.2) belonging to the AA1 family (multi-copper oxidases) has conserved motifs (copper binding motifs) within its amino acid sequence, namely His-Xaa-His-Gly (HXHG), His-Xaa-His (HXH), His-Xaa-Xaa-His-Xaa-His (HXXHXH), and His-Cys-His-Xaa^3^-His-Xaa^4^-Met/Leu/Phe (HCHXXXHXXXXM/L/F) [68]. We compared the sequences of genes predicted to encode the laccase (AA1) with those of previously reported *F. velutipes* laccase genes, including *fvLac-1* (KM276550), *fvLac-2* (KM276551), *fvLac-3* (KM276552), and *fvLac-4* (KM276553) [69]. Our results showed that the predicted laccase (AA1 family) gene shared 52.5–98% amino acid sequence similarities with those of previously reported *F. velutipes* laccase genes [69] and contained the same four copper-binding motifs (Figure S1B). These results suggest that the gene predicted to encode AA1 may act as a laccase involved in depolymerization of a non-carbohydrate structural component (lignin). Furthermore, GMC oxidoreductase proteins (AA3 family) have been reported to possess a β-α-β dinucleotide binding-motif consisting of Gly-Xaa-Gly-Xaa-Xaa-Gly-Xaa^18^-Glu (GXGXXGX^18^E), which interacts with the flavin adenine dinucleotide cofactor [70,71,72]. *F. elastica* was also found to contain 36 putative GMC oxidoreductases assigned to family AA3 (Table S10). Among the 36 genes, 17 genes contained the same β-α-β dinucleotide-binding motif (Figure S1C), suggesting that the enzymes encoded by those genes are most likely to act as GMC oxidoreductases.

The process of converting biomass (mainly carbohydrates) into biofuels, such as bioethanol, is known [73,74]. However, plant cell walls often form complex structures and contain a significant amount of lignin, which is typically considered an obstacle to producing bioethanol because of the difficulty of depolymerizing this structural component. However, several reports have suggested that microbial enzymatic strategies could be used to degrade the recalcitrant lignin matrix [53,75,76]. Generally, wood degradation by white rot fungi starts with the depolymerization of lignin, which leads to further degradation of nearby wood polymers by highly reactive lignin radicals [77,78]. Our results indicate the extensive range of enzymes that belong to AA families in the *F. elastica* genome, suggesting the strong potential for this white rot fungus to be used for biomaterial and bioenergy production in the future.

## 3. Materials and Methods

### 3.1. Fungal Strain Culture and Genomic DNA Isolation

*Flammulina elastica* KACC46182 was obtained from the National Agrobiodiversity Center (http://genebank.rda.go.kr/) and was grown at 26 °C on mushroom complete medium (MCM) agar (0.2% peptone, 2% glucose, 0.2% yeast extract, 0.05% MgSO_4_, 0.046% KH_2_PO_4_, 0.1% K_2_HPO_4_, and 1.5% agar) for 14 days. Genomic DNA was isolated from *F. elastica* as described by Park et al. [8]. Briefly, DNA extraction buffer (100 mM NaCl, 50 mM ethylenediaminetetraacetic acid, 0.25 M Tris-HCl, 5% SDS), 2 × CTAB buffer (2% CTAB, 100 mM Tris-HCl pH 8.0, 20 mM EDTA pH 8.0, 1.4 M NaCl, 1% polyvinyl pyrrolidone), and phenol-chloroform-isoamylalcohol (25:24:1) were added to fresh mycelia and briefly vortexed. After 5 min of incubation at room temperature, samples were centrifuged at 13,000 rpm at 4 °C for 5 min. Supernatants were mixed with 0.7 volumes isopropanol and centrifuged for 10 min at 4 °C. After washing with 70% ethanol, air-dried samples were eluted in TE buffer and treated with RNase A (Qiagen, Seoul, Korea).

### 3.2. Genome Sequencing and De Novo Assembly

Next-generation sequencing (NGS)-based genome sequencing of the *F. elastica* genome was performed using a HiSeq 2000 platform (Illumina, Inc., San Diego, CA, USA) according to the manufacturer’s protocol. All sequencing data were analyzed for quality control using FastQC (http://www. bioinformatics.babraham.ac.uk/projects/fastqc/) and further processed using Trimmomatic (version 0.32) [79] to remove bad quality reads and sequencing adapters. The resulting short reads were used for assembly using Velvet Optimiser [80] with a kmer-size search range of 17–31.

### 3.3. Gene Prediction and Annotation

Ab initio gene structure prediction was carried out using the AUGUSTUS tool [81], trained with *Laccaria bicolor*. For functional annotation, the predicted genes of *F. elastica* were compared with the National Center for Biotechnology Information (NCBI) non-redundant database and fungal genome database using DIAMOND [82] and BLASTP (version 2.2.31) software. In addition, a protein family search was conducted against the protein family database (Pfam 31.0, http://pfam.xfam.org) with Pfam-scan software [83]. tRNAscan-SE (version 2.0) software [12] was used to predict tRNA in the *F. elastica* genome.

### 3.4. Ortholog Identification and Clustering

The predicted genes (proteins) of *F. elestica* were clustered into orthologous groups using OrthoFinder (version 2.2.1) software [84]. Orthologs were identified and clustered by an all-versus-all protein comparison with predicted proteins of the following fungal species; *A. nidulans* FGSC-A4 [25], *Botrytis cinerea* B05.10 [48], *Agaricus bisporus* var. *bisporus* H97 [85], *C. cinerea* okayama7#130 [43], *C. militaris* CM01 [26], *C. neoformans* var. grubii H99 [59], *F. velutipes* KACC42780 [11], *L. bicolor* S238N-H82 [67], *L. edodes* [66], *Neurospora crassa* OR74A [86], *P. chrysosporium* RP78 [9], *S. cerevisiae* S288C [27], *S. commune* H4-8 [47], *T. reesei* QM6a [28], and *U. maydis* 521 [10]. 

### 3.5. CAZyme Gene Identification and Signal Peptide Prediction

CAZymes, including those encoded by GH, GT, PL, CE, and AA genes in *F. elastica* and 10 other sequenced fungal species were identified and annotated using the HMMER 3.0 package (http://hmmer.org/) with the dbCAN CAZyme database (http://csbl.bmb.uga.edu/dbCAN/) [13]. Prediction of signal peptides in the CAZyme genes was conducted using the SignalP 4.1 server (http://www.cbs.dtu.dk/services/SignalP/) [87].

### 3.6. Data Access

Sequence reads were deposited in the Sequence Read Archive (SRA) at NCBI under the following accession number: SRP151642.

## 4. Conclusions

This study aimed to advance the understanding of the lignocellulolytic machinery in the mushroom-forming basidiomycete fungus *F. elastica* for biotechnological and industrial applications. Recently, *F. velutipes* was found to efficiently convert glucose to ethanol, similar to *S. cerevisiae* [88,89]. *F. velutipes* was also found to convert cellobiose, cellotetraose, cellotriose, maltose, and sucrose to ethanol, with similar recovery rates as that of glucose. These capabilities of *F. velutipes* can be applied to bioethanol production processing, which is known as consolidated bioprocessing (CBP). CBP is considered an effective alternative to high-cost biomass processing for bioethanol production from lignocellulosic biomass [90,91,92]. In our previous study, we found that *F. velutipes*, the closest white rot fungus to *F. elastica*, is a highly attractive model for bioethanol production because of its highly developed lignocellulolytic machinery, as well as its vast array of genes associated with ethanol production [11]. In the present study, we conducted sequencing of the *F. elastica* genome to identify the machinery involved in lignocellulosic biomass degradation. As described above, many CAZyme genes were identified in the *F. elastica* genome including 218 GHs, 18 PLs, 59 CEs, and 82 AAs associated with polysaccharide and lignin degradation (Figure 3). From the genome sequence of *F. elastica*, 318 more genes were predicted than *F. velutipes*, but *F. velutipes* were found to have 32 more CAZymes (Table 2 and S7). However, in ortholog analysis between *F. elastica* and *F. velutipes*, 22 genes associated with CAZymes were found only in the genome of *F. elastica*. In addition, the distribution of these CAZyme genes in *F. elastica* was comparable to those of other wood-rotting basidiomycetes, and there were more than those in the model white rot fungus *P. chrysosporium* [9] (Figure 3). Although further detailed investigations of CAZyme genes are needed, the present study suggests that *F. elastica* holds great potential for future biomaterial and bioenergy production.

## Figures and Tables

**Table 1:** *Flammulina elastica* Genome Sequencing Statistics.

**Hiseq 2000 NGS Analysis**	Total reads (100 bp)	57,658,112
	Reads after trimming (%), >Q 30	53,555,252 (92.88)
**Velvet De Novo Assembly**	Optimized Velvet hash value (kmer)	31
	Total number of contigs	13,877
	Number of contigs (>1 kb)	2055
	Contig N50 (bp)	42,684
	Length of longest contig (bp)	275,350
	Total bases in contigs (bp)	35,045,521
	Total bases in contigs (>1 kb)	33,024,561
	GC content (%)	49.85
**Gene Prediction**	Predicted gene	12,536
	Average gene length (bp)	1973
	Average protein length (aa)	524.34
	Average exon per gene	6.72
	Average exon size (bp)	233.91
	Average intron size (bp)	69.29

**Table 2:** Comparison of the Genome Characteristics of *Flammulina elastica* and other Basidiomycetes.

Fungal Species	*F. elastica*	*F. velutipes*	*L. bicolar*	*C. cinerea*	*P. chrysosporium*	*U. maydis*	*S. commune*
**Strain**	KACC46182	KACC42780	S238N-H82	Okayama7#130	RP78	521	H4-8
**Genome Assembly (Mb)**	35	35.6	64.9	37.5	35.1	19.7	38.5
**Number of Protein-Coding Genes**	12,536	12,218	20,614	13,544	10,048	6522	13,181
**GC Contents (%)**	49.7	48.99	46.6	51.6	53.2	54.0	56.6
**Average Gene Length (bp)**	1973	2294	1533.0	1679.0	1667.0	1935.0	1794.9
**Average Exon Size (bp)**	233.91	231.4	210.1	251.0	232.0	1051.0	249.3
**Average Intron Size (bp)**	69.29	190.3	92.7	75.0	117.0	127.0	79.0

## Supplementary Materials

The following figures are available online at http://www.mdpi.com/1422-0067/19/8/2379/s1.
